# Supplementary material for: Strengthening in multi-principal element alloys with local-chemical-order roughened dislocation pathways
Source: Nat Commun. 2019 Aug 8;10:3563. doi: 10.1038/s41467-019-11464-7 (PMC6687833; doi:10.1038/s41467-019-11464-7)
Supplement: Supplementary file 1 — Supplementary Information [file 41467_2019_11464_MOESM1_ESM.pdf]

Supplementary Information

Strengthening in multi-principal element alloys with local-chemical-order roughened dislocation pathways

Li et al.

Supplementary Note 1

Supplementary Table 1 | EAM potential predicted properties for Ni, Co, Cr, in comparison with experiments (or ab initio calculations), where E_c is the cohesive energy, C_{11}, C_{12} and C_{44} elastic constants, ν phonon frequencies.

Ni	EAM	Experiment / Theory
E_c (eV/atom): fcc, 300K, a = 3.52 Å	-4.45	-4.45[a]
C_{11} (GPa)	262	262[b]
C_{12} (GPa)	155	150[b]
C_{44} (GPa)	122	131[b]
$\nu_L(X)$ (THz) (fcc a = 3.52 Å)	8.57	8.55[c]
$\nu_T(X)$ (THz) (fcc a = 3.52 Å)	6.18	6.27[c]
$\Delta E_{fcc-hcp}$ (eV/atom)	0.03	0.03[d]
$\Delta E_{fcc-bcc}$ (eV/atom)	0.10	0.09[d]
**Co**	**EAM**	**Experiment / Theory**
E_c (eV/atom): hcp, 300K, a = 2.507 Å, c = 4.069 Å	-4.39	-4.39[a]
C_{11} (GPa) (fcc a = 3.53 Å)	260	259[b]
C_{12} (GPa) (fcc a = 3.53 Å)	165	159[b]
C_{44} (GPa) (fcc a = 3.53 Å)	102	109[b]
$\nu_L(X)$ (THz) (fcc a = 3.53 Å)	8.06	8.1[c]
$\nu_T(X)$ (THz) (fcc a = 3.53 Å)	5.75	5.8[c]
$\Delta E_{fcc-hcp}$ (eV/atom)	0.014	0.016[d]
$\Delta E_{fcc-bcc}$ (eV/atom)	0.12	0.13[d]
**Cr**	**EAM**	**Experiment / Theory**
E_c (eV/atom): bcc, 300K, a = 2.91 Å	-4.10	-4.10[a]
C_{11} (GPa) (bcc a = 2.91 Å)	380	391[b]
C_{12} (GPa) (bcc a = 2.91 Å)	161	89[b]
C_{44} (GPa) (bcc a = 2.91 Å)	77	103[b]
ν (X) (THz) (bcc a = 2.91 Å)	6.5	7.8[c]
ν (P) (THz) (bcc a = 2.91 Å)	7.94	8.2[c]
$\Delta E_{bcc-fcc}$ (eV/atom)	0.22	0.4[d]
$\Delta E_{bcc-hcp}$ (eV/atom)	0.22	0.46[d]

[a]Ref. 1; [b]Ref. 2; [c]Ref. 3; [d]ab initio calculation in the present work.

Note: the as-developed Ni-Co-Cr EAM potential is available upon request.

The predicted physical properties from our newly developed NiCrCo EAM (embedded atom method) potential are compared with the experimental/DFT values in Supplementary Table 1-2 and Supplementary Figure 1-4. Very good performance has been achieved for pure elements,

intermetallic compounds and complex solid solutions. More discussions on magnetic effects are presented in Supplementary Note 9.

Supplementary Table 1 lists the basic properties of elemental structures calculated from our EAM potential and the corresponding experimental/theoretical values. Supplementary Figure 1 shows the equations of state of different allotropes of the elements. The EAM potential can correctly predict the ground states of the elements in accordance with ab initio calculations. For Co, the cohesive energies of face-centered-cubic (FCC) and hexagonal close packed (HCP) structures are very close, with FCC Co being the ground state in the non-spin polarized DFT calculation. Supplementary Figure 2 shows the predicted cohesive energies for the three binary systems involved. The energies and lattice constants of the energetically optimized structures are also provided in Supplementary Table 2. The energy differences between EAM and ab initio calculations (Supplementary Table 2) are within several tens of meV, which is indicative of a high-quality interatomic potential for metallic alloys.

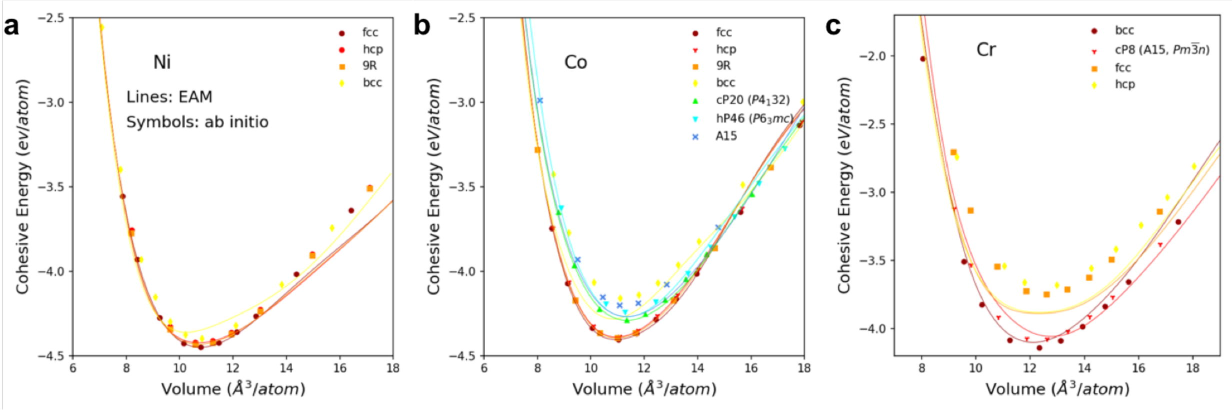

Supplementary Figure 1 | Equations of state of selected crystal structures. **a**, Equation of state for Ni. **b**, Equation of state for Co. **c**, Equation of state for Cr.

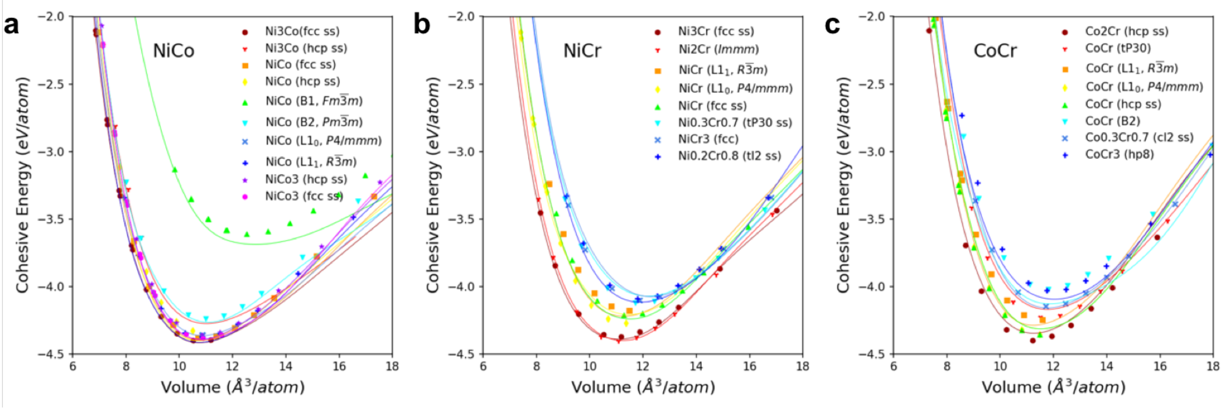

Supplementary Figure 2 | Comparisons of *ab initio* and EAM calculations of the cohesive energies of different intermetallics. **a**, Ni-Co intermetallics. **b**, Ni-Cr intermetallics. **c**, Co-Cr intermetallics.

Supplementary Table 2 | Comparisons between EAM predications and ab initio evaluations of the cohesive energy and lattice parameters of selected intermetallic compounds for the three binary systems. SS: solid solution.

		EAM		Ab initio	
**Composition**	Structure	Lattice parameter (Å)	E_c (eV/atom)	Lattice parameter (Å)	E_c (eV/atom)
**Ni_3Co**	FCC SS	3.506	-4.419	3.510	-4.407
**NiCo**	FCC SS	3.514	-4.402	3.516	-4.385
**NiCo**	B1, $Fm\bar{3}m$	3.710	-3.688	3.690	-3.630
**NiCo**	B2, $Pm\bar{3}m$	2.810	-4.265	2.801	-4.240
**NiCo**	$L1_0$, $P4/mmm$	a = 3.522<br>b = 3.503	-4.365	a = 3.530<br>b = 3.553	-4.366
**NiCo**	$L1_1$, $R\bar{3}m$	v = 10.748 Å^3	-4.408	v = 10.8565 Å^3	-4.374
**$NiCo_3$**	FCC SS	3.519	-4.395	3.519	-4.385
**Ni_2Cr**	$Immm$	a = 2.496<br>b = 3.600<br>c = 7.50	-4.399	a = 2.484<br>b = 3.583<br>c = 7.463	-4.404
**Ni_3Cr**	FCC SS	3.546	-4.390	3.553	-4.383
**NiCr**	$L1_1$, $R\bar{3}m$	v = 11.292 Å^3	-4.221	v = 11.520 Å^3	-4.190
**NiCr**	$L1_0$, $P4/mmm$	a = 3.599<br>b = 3.579	-4.210	a = 3.595<br>b = 3.576	-4.265
**NiCr**	FCC SS	3.584	-4.242	3.559	-4.212
**$Ni_{0.3}Cr_{0.7}$**	SS., $P4_2/mnm$	a = 8.910<br>b = 4.643	-4.075	a = 8.682<br>b = 4.530	-4.118
**$NiCr_3$**	FCC SS	3.667	-4.076	3.624	-4.103
**$Ni_{0.2}Cr_{0.8}$**	BCC SS	2.876	-4.116	2.868	-4.093
**Co_2Cr**	HCP SS	a = 2.525<br>c = 4.065	-4.347	a = 2.527<br>c = 4.069	-4.405
**CoCr**	$L1_1$, $R\bar{3}\,m$	v = 11.263 Å^3	-4.290	v = 11.608 Å^3	-4.261
**CoCr**	SS, tP30, $P4_2/mnm$	a = 8.785<br>c = 4.553	-4.172	a = 8.737<br>c = 4.528	-4.231
**CoCr**	$L1_0$, $P4/mmm$	a = 3.587<br>b = 3.568	-4.316	a = 3.592<br>b = 3.572	-4.315
**CoCr**	HCP SS	a = 2.549<br>c = 4.103	-4.317	a = 2.543<br>c = 4.094	-4.362
**CoCr**	B2, $Pm\bar{3}m$	2.872	-4.124	2.870	-4.016
**$Co_{0.3}Cr_{0.7}$**	BCC SS	2.868	-4.160	2.875	-4.150
**$CoCr_3$**	hP8, $P6_3/mmc$	a = 5.183<br>c = 4.158	-4.094	a = 5.130<br>c = 4.116	-4.040

Now we consider the performance of our EAM potential on the ternary NiCoCr solid solutions. The as-obtained potential has been tested for a number of physical properties of NiCoCr medium-entropy alloys. We first show the performance of the potential in describing NiCoCr alloys in a large phase space. Supplementary Figure 3 shows the comparisons of the energies of NiCoCr solid solutions estimated using EAM and ab initio treatments. As seen, the EAM results are highly consistent with the non-spin polarized DFT calculation results, suggesting a high accuracy of our EAM potential.

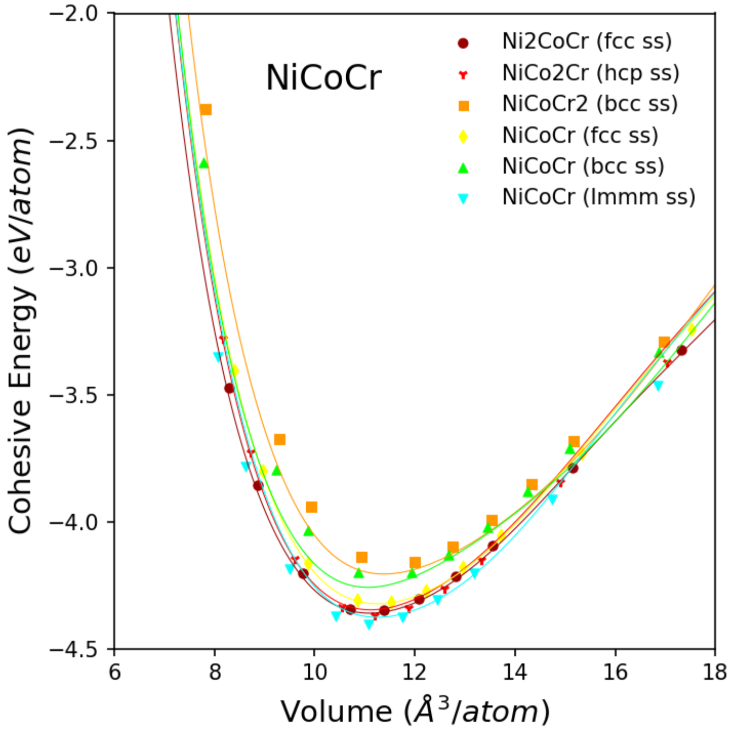

Supplementary Figure 3 | The cohesive energies of selected NiCoCr solid solutions as a function of volume.

The as-developed potential has been utilized to evaluate the physical properties of NiCoCr solid solutions in the entire compositional range, which would otherwise be impossible with the DFT calculations, as shown in Supplementary Figure 4. During our calculation, a large system containing 72,000 atoms with the desired composition was created with atoms randomly arranged on an FCC lattice, followed by conjugated-gradient energy minimization to find the lowest energy minimum. The elastic constants were analytically calculated based on the second-derivatives of the potential energy. It can be seen that the EAM potential is applicable to study FCC NiCoCr in the entire compositional range, and all the configurations are found to be mechanically stable based on the Born criterion $C_{11} - C_{12} > 0$ and $C_{44} > 0$. In terms of cohesive energy and lattice constant, it is found that both quantities can be described by the rule of mixtures for FCC NiCoCr solid

solutions, where the deviations of the cohesive energy and the lattice constant from the rule of mixtures are within 1.2% and 0.3%, respectively.

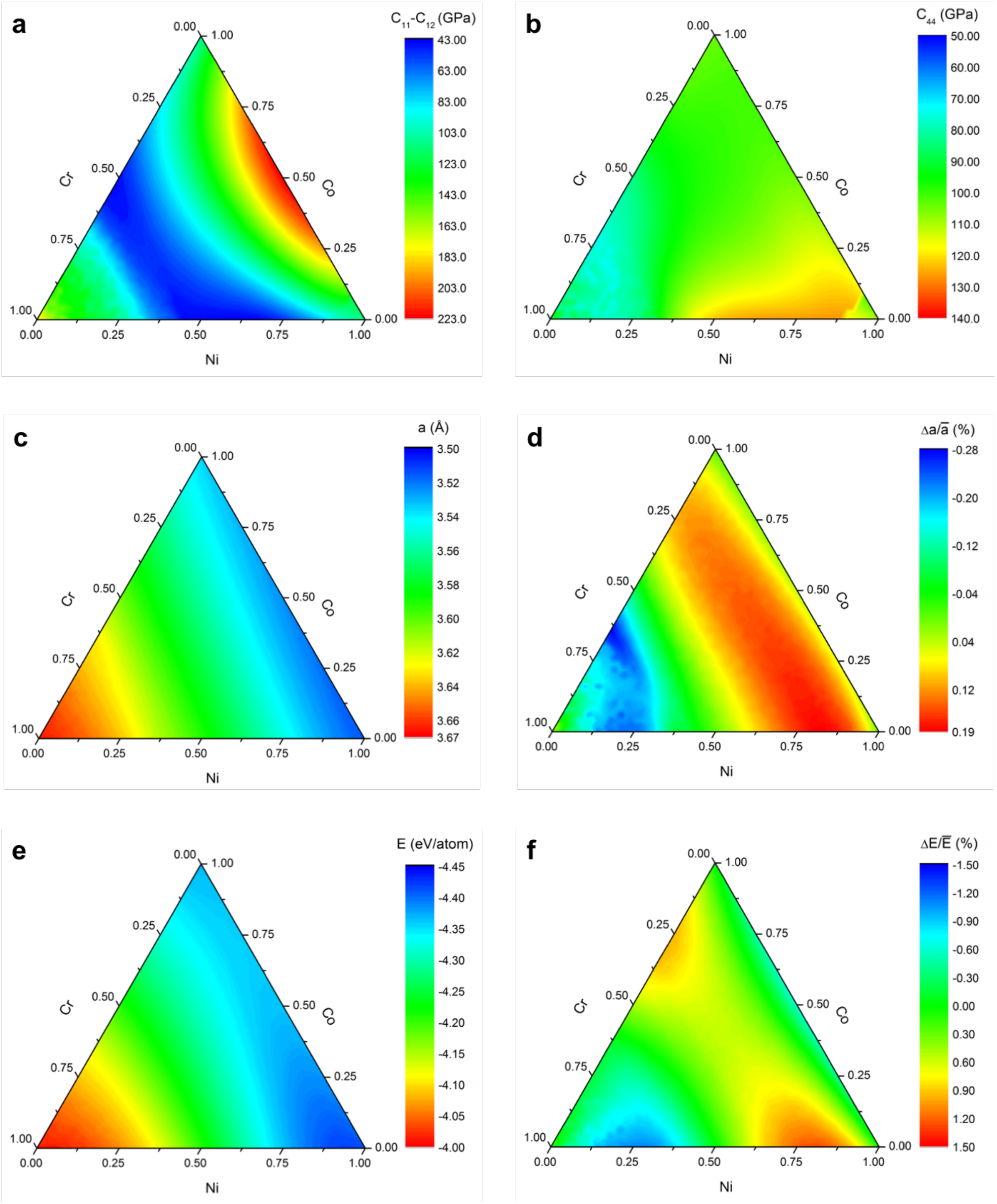

Supplementary Figure 4 | EAM predictions on physical properties of NiCoCr alloys in the entire compositional range. **a-b**, FCC phase of NiCrCo in the entire composition region is mechanically stable, based on the Born criterion $C_{11} - C_{12} > 0$ and $C_{44} > 0$. **c**, lattice constants a. **d**, deviations of calculated a from that of rule of mixture. **e**, cohesive energies E_c. **f**, deviations of calculated E_c from that of rule of

mixture. **c-f** suggest that Vegard's law is obeyed by the Ni-Co-Cr solid solutions, i.e., the *a* (E_c) of an FCC NiCoCr solid solution is approximately equal to the geometric mean of the constituents' a (E_c), largely satisfying the rule of mixture.

We also calculated the generalized stacking fault energy (GSFE) of random NiCoCr alloy (Supplementary Figure 5), using the newly developed EAM potential. We constructed random solid solutions of equi-atomic NiCoCr alloy, with the [111] direction aligned along the *z*-axis. Periodic boundary conditions were only applied in the *x*, *y* directions (a similar method has been adopted in our previous work[2]). The size of the *x*-*y* plane is set to 12.42 × 12.92 Å^2, which is close to the critical size for dislocation nucleation under certain stress and temperature conditions[3]. The upper half of the crystal was displaced along the $[11\bar{2}]$ direction on the (111) slip plane. The GSFE line shape was obtained by conducting statistical GSFE analysis on >10,000 random configurations. The position dependent intensity indicates the probability for a GSFE line to be located. For a specific cross-section along the line, we can also derive the distributions of the fault energies, similar to that shown in Fig. 3 of the main text. For example, the cross-section for intrinsic stacking fault energy suggests a wide distribution of the intrinsic stacking fault energy with a mean negative value, which is consistent with previous work[2,4], further validating the accuracy of our EAM potential.

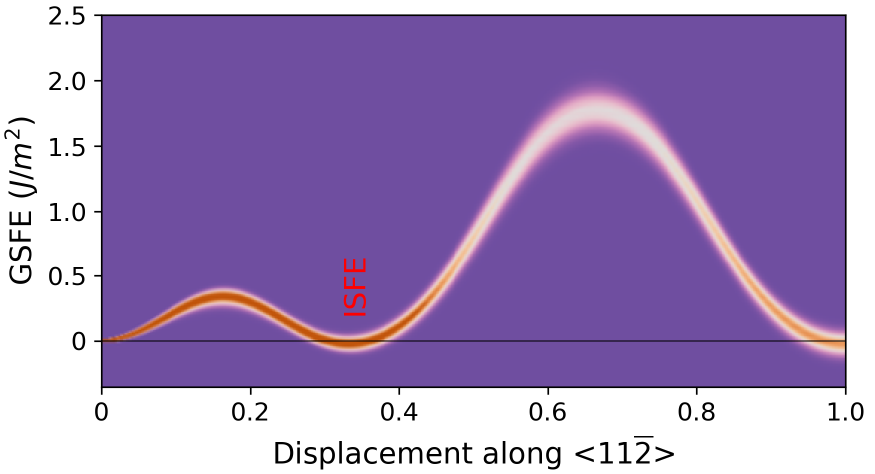

Supplementary Figure 5 | Line broadening of generalized stacking fault energy curve for random solid solutions at 0 K. The stacking fault energy has a finite distribution, which is a hallmark signature of HEAs. The wide distribution of the stacking faults has important implications for the mechanical properties of the chemically disordered crystal. The unit length along the displacement direction is $a\sqrt{6}/2$, where *a* is the lattice constant.

Within the context of stacking fault energy (SFE), most of the previous studies of HAEs concern the average value of SFE, where the error bars are associated with uncertainties in experimental measurements. Here, by "line broadening", we refer to a wide statistical distribution

of the SFE that exhibits a broadened line profile (an intrinsic feature of HAE rather than uncertainties from measurements). It contains more information than an average SFE value plus an error bar. The line profile of the SFE distribution has a significant impact on the deformation behavior of the HAE, as discussed in the main text.

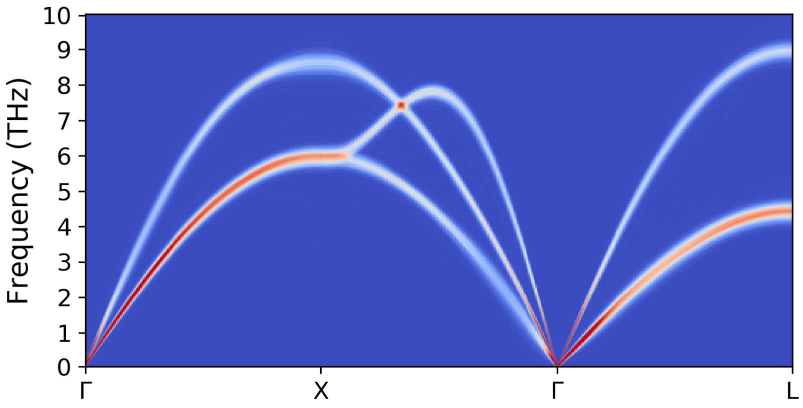

Supplementary Figure 6 | Phonon dispersion line broadening in homogeneously disordered NiCoCr MEA due to mass disorder and force-constant disorder, obtained with the EAM interatomic potential.

Line-broadening of the phonon dispersion curves has been known for homogeneously disordered solid solutions[5,6], such as Fe-Cr[7], due to mass disorder and force-constant disorder. There is also increasing evidence to show that the local chemical order (LCO) has an effect on the lattice dynamics, hence the stability of the crystals[5]. Correct interpretation and prediction of the lattice dynamics of HAE are therefore important. Theoretically, the phonon dispersions of random solid solutions can be treated with different approaches, e.g., the coherent crystal approximation[5] and the special quasi random structures (SQS) method[8]. However, as far as the line shape of the phonon spectra of disordered alloys is concerned, the calculations are much more involved. Here we use a different method to calculate the line broadening of the phonon dispersion curves based on the empirical potential.

For any given lattice parameter and wave vector, the phonons can be computed by diagonalizing the dynamical matrix according to[9]

$$D_{\lambda\mu}(\mathbf{q}) = \frac{1}{\sqrt{m_i m_j}} \sum_{lk} \Phi_{\lambda l \mu k} \ \exp\left[i\mathbf{q} \cdot (\mathbf{R}_l - \mathbf{R}_k\right] \quad (1)$$

where i and j are particle indices; m_i and m_j are masses of particle i and j; α and β are force components (x, y, or z); $\lambda = 3i + \alpha$ and $\mu = 3j + \beta$. The summation over l, k represents the sum over lattice vectors $\mathbf{R_l}$, $\mathbf{R_k}$ within the cutoff radius. $\Phi_{\lambda l \mu k}$ is the force constant.

In this work, we used the temperature-dependent effective potential method (TDEP)[10] to obtain the effective interatomic force constants (IFCs) of a supercell (108 atoms) of NiCoCr

random solid solution. Classical MD simulations were carried out at 300 K to derive atomic displacements and atomic forces. The IFCs were optimized with the *alamode* code[11] based on the force and displacement relationship. Having obtained the IFCs, the dynamical matrix was derived by assuming an averaged atomic mass occupying the FCC unit-cell following the above equation. This method provides a rapid route to map out the phonon dispersion, enabling the estimate of the line shape of the phonon dispersion curves as shown in Supplementary Figure 6 (collected over 50,000 different atomic configurations of random NiCoCr). As far as the phonons are concerned, random solid solutions represent a special type of crystal where the lattice periodicity is well defined (this is different from amorphous alloys), as evidenced from the X-ray diffraction patterns of solid solutions. In the reciprocal space, there is a full Brillouin zone associated with the lattice. This is the reason why random solid solutions can be treated with virtual crystal approximation (VCA) in first principles calculations. Experimentally, the phonon dispersion curves can be measured by various techniques such as inelastic x-ray scattering. Phonon line broadening of 12 bcc HAEs was recently investigated with ab initio treatments[6].

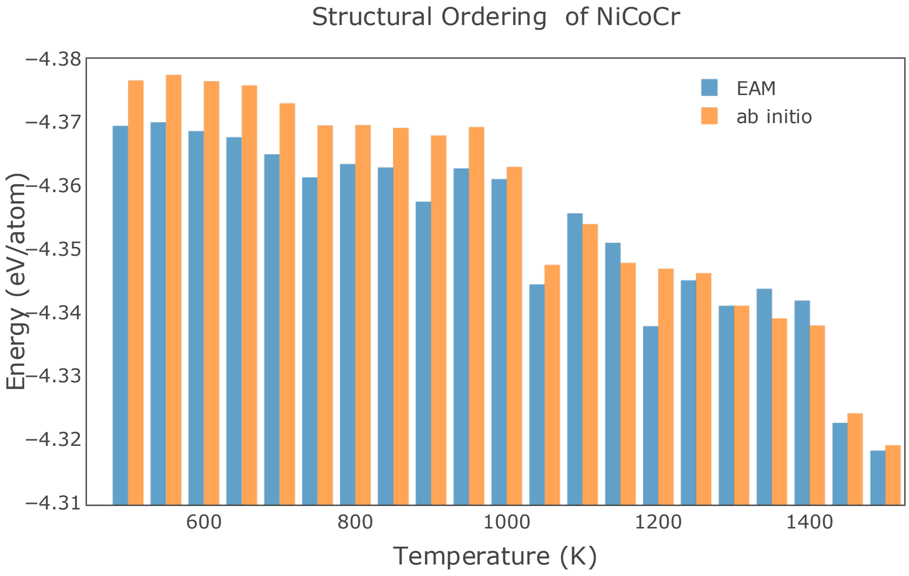

Supplementary Figure 7. Comparisons of the energies of NiCoCr alloy with different degrees of chemical ordering by EAM and ab initio calculations respectively. The chemical ordering in the alloy was achieved by conducting hybrid MD/MC simulations in a 360-atom ensemble quenched from 1500 K, employing the as-developed empirical EAM potential.

We show in Supplementary Figure 7 that the EAM potential yields a cohesive energy trend with increasing chemical ordering, consistent with the DFT calculations. This suggests that our EAM potential is capable of predicting the correct trend of potential energy changes due to chemical ordering during the annealing process.

Chemical ordering is a common phenomenon associated with concentrated solid solutions[12], such as Fe-Cr[13] and Ni-Cr[14]. How to correctly predict and characterize the ordering states in these alloys under different thermodynamic conditions remains a long-standing materials science problem. The chemical ordering of different species is dictated by the thermodynamics of the system, which is a manifestation of interatomic interactions. Hence, the prediction of chemical ordering would require accurate descriptions of the atomic interactions, which, however, is highly challenging for multicomponent systems because many different types of atomic pairs are involved, and the many-body type interactions are complex. As such, the correct trend of chemical ordering may serve as a reliability test for the validation of the interatomic potentials.

To study the chemical ordering process in the NiCoCr alloy, we are primarily concerned with two questions: (1) whether chemical ordering is going to happen, and (2) what type of chemical ordering is expected if it is kinetically permitted.

Employing the newly developed NiCoCr EAM potential, we carried out hybrid MD/MC simulations to investigate the chemical ordering in equi-atomic NiCoCr alloy cooled down from high temperatures. At each temperature, the atoms in the system were swapped periodically based on the Metropolis algorithm to facilitate atomic diffusion kinetics. Naturally, the system would evolve toward more thermodynamically stable states with different degrees of chemical order. To better assess the energy states of the atomic configurations, we conducted energy minimization to remove the thermal vibrations. The energies of the as-obtained configurations were subjected to high-precision *ab initio* calculations for cross-checks. The comparisons of the energies of the chemically ordered structures calculated using EAM and ab initio treatments are shown Supplementary Figure 7.

It can be seen that the energy trend of "ordered states" predicted by EAM agrees with that from ab initio calculations, that is, the cohesive energy decreases with increasing chemical ordering, suggesting that the as-developed NiCrCo potential is capable of capturing the increasing stabilities with increasing chemical ordering.

Supplementary Note 2

The chemical short-range order parameters α^2 and α^3 for different element-pairs are shown in Supplementary Figure 8 for a wide range of annealing temperatures. For the second nearest neighbor shell (SNNS), Ni appears to be segregated at all T_a. Co-Co and Cr-Cr chemical ordering experiences an obvious transition around 750 K, i.e., Co-Co pairs and Cr-Cr pairs are not favored in the SNNS above 750 K; but the trend is reversed when $T_a \leq 750$ K, consistent with the formation of more ordered Co-Cr domains shown in Fig. 1 of the main text. The Co-Cr interactions of the SNNS remain similar to those of the first nearest neighbor shell (FNNS), i.e., it tends to form Co-

Cr neighbors. Other Ni-related chemical orders remain similar to that for FNNS. For the third nearest neighbor shell (TNNS), the Co-Cr interactions still remain strong, i.e., it tends to form Co-Cr clusters in the TNNS. Co, Cr and Ni all tend to attract the same species in this TNNS. Ni-Co and Ni-Cr pairs are not favored in the TNNS.

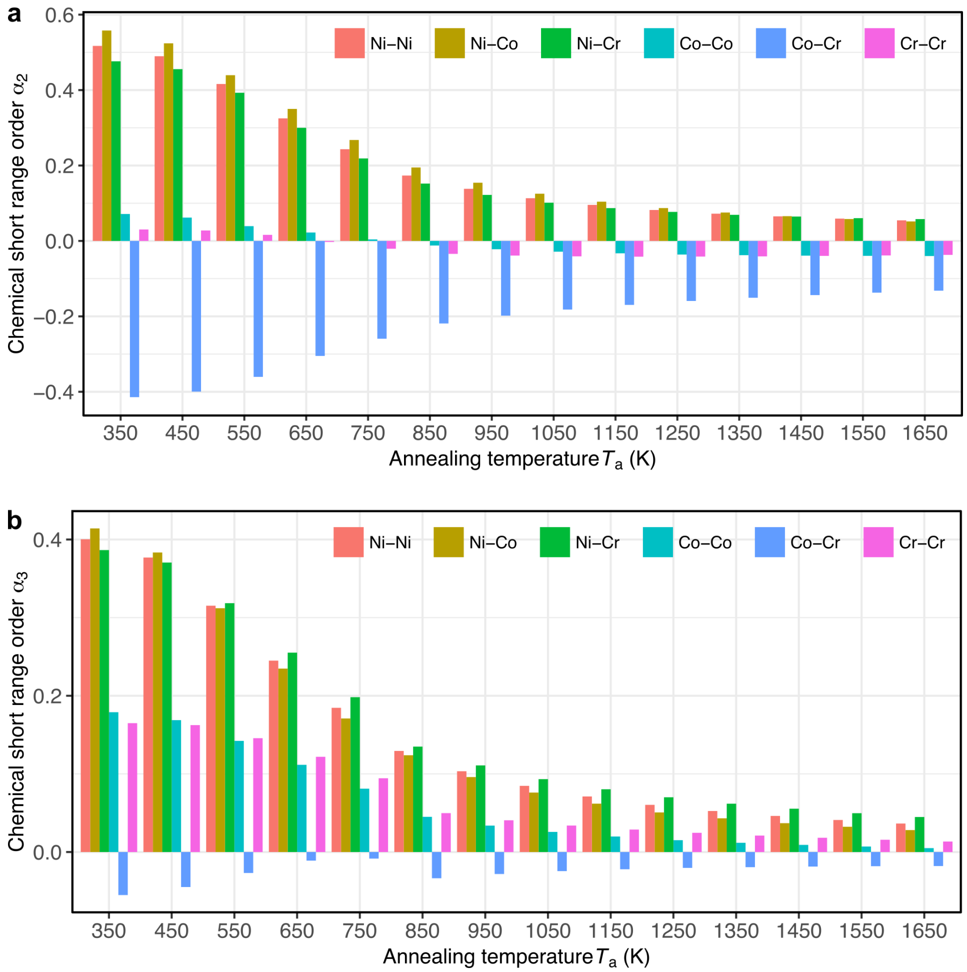

Supplementary Figure 8 | Chemical short-range order parameter α^2 (**a**) and α^3 (**b**) at different annealing temperatures.

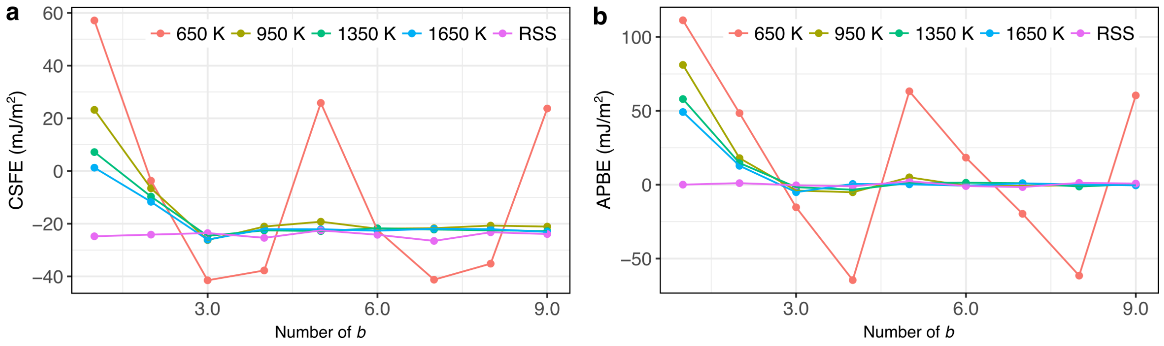

Supplementary Figure 9 | Effects of increasing slip amount on fault energies. **a**, Variations of complex stacking fault energy with increasing number of Burgers vector *b* on the same slip plane. **b**. Variations of anti-phase boundary energy with increasing amount of slip. Temperatures in the legends means annealing temperature while all the calculations are carried out at 0 K.

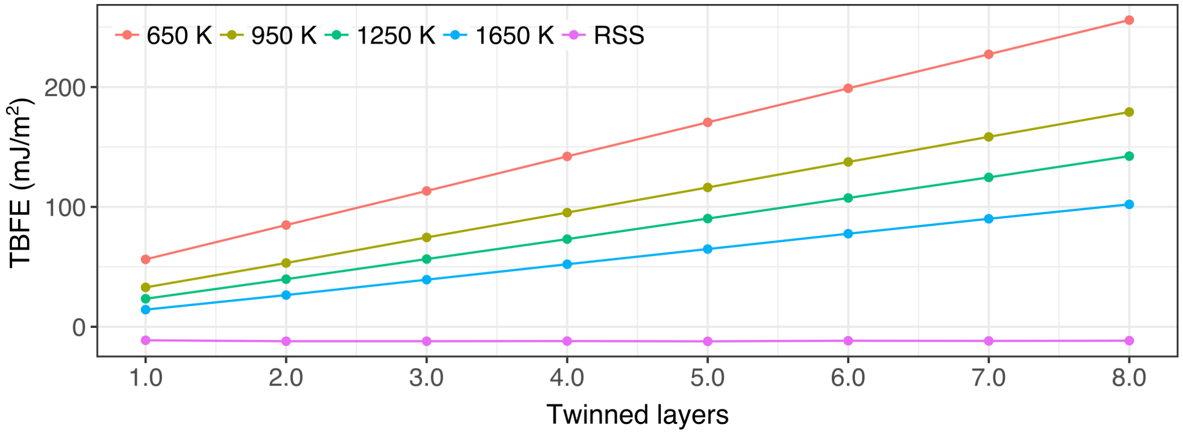

Supplementary Figure 10 | Variations of twin boundary fault energy with respect to twinned layers. Temperatures in legends are the annealing temperature. All twin boundary energy calculations are performed at 0 K.

Supplementary Note 3

For basic property assessments, the dimensions of the sample are 10 nm × 10 nm × 10 nm, along the [100], [010] and [001] directions, respectively. Periodic boundary conditions are applied in all directions. All samples were annealed at each annealing temperature using the hybrid MD/MC simulations until the LCO converges. Each property at a specific annealing temperature was calculated at zero temperature and averaged over ten configurations equally distributed over the last one million MD/MC steps. The calculated elastic properties include C_{11}, C_{12}, C_{44}, bulk modulus B and Poisson's ratio ν. In Supplementary Figure 11, C_{11} is the average value of C_{11}, C_{22} and C_{33} of the cubic sample. C_{12} is the average value of C_{12}, C_{13}, and C_{23} of the cubic sample. C_{44}

is the average value of C_{44}, C_{55} and C_{66} of the cubic sample. As seen in Supplementary Figure 11a, generally, the elastic properties slightly increase with increasing LCOs except for a critical annealing temperature range where significant chemical ordering develops. The fluctuations at the critical annealing temperature range might be due to the anisotropies developed in elastic constants, as a result of significant chemical ordering. For example, the tensor components C_{11}, C_{22} and C_{33} may no longer be equal in magnitude, i.e., some of them become larger while some of them become relatively smaller such that the average is even smaller than that of a sample with weaker LCOs. However, with further increasing LCOs, the magnitudes of all components continue to increase despite that the anisotropy still exists, resulting in increasing average values again. As seen in Supplementary Figure 11b, with increasing LCOs, the average lattice constant slightly increases while the cohesive energy gradually decreases, suggesting a trend toward more stable states.

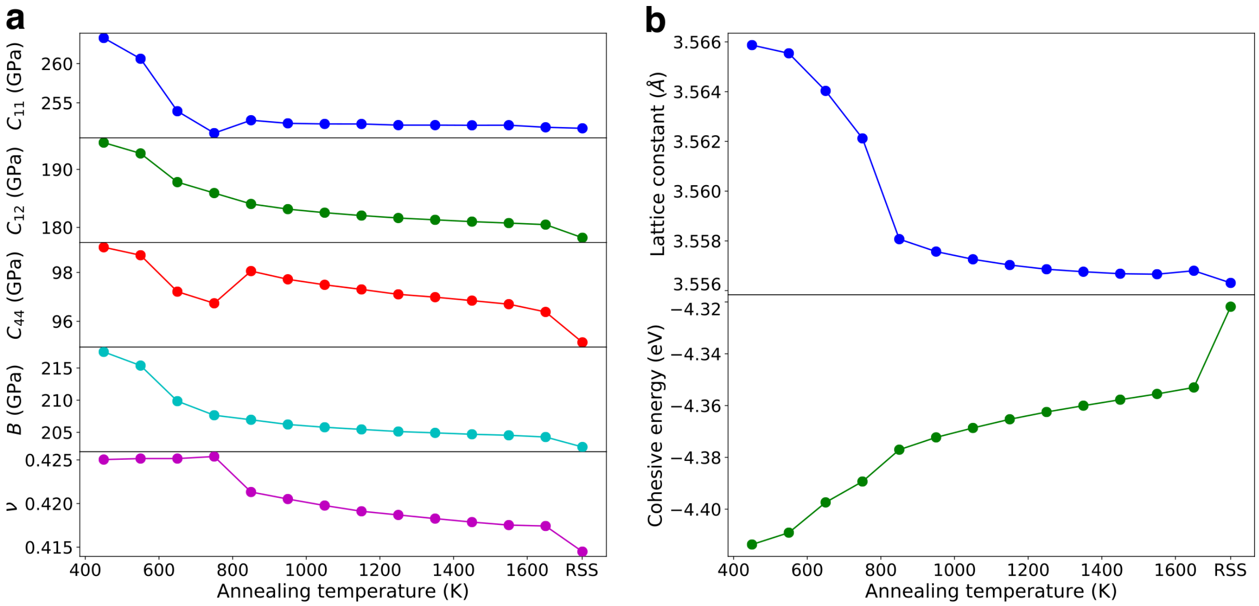

Supplementary Figure 11 | Annealing temperature dependent material properties. a, Elastic properties. **b,** Lattice constant and cohesive energy.

Supplementary Note 4

Supplementary Figure 12 shows the nanoscale property mapping, in a sample with T_a = 650 K, to correlate local properties with LCOs. Supplementary Figure 12a shows the element distributions and the corresponding α_{ij}^{1} distributions. As expected, local composition fluctuations correspond to obvious spatial variations in α_{ij}^{1}. However, no strong correlations are seen between these α_{ij}^{1} distributions and the distributions of local CSFEs (Supplementary Figure 12b, leftmost panel) and local APBEs (Supplementary Figure 12c, leftmost panel). Instead, the change of certain α_{ij}^{1} after

introducing the fault, i.e., $\Delta\alpha_{ij}^{1} = \alpha_{ij}^{1,\text{fault}} - \alpha_{ij}^{1,\text{perfect}}$, correlates well with the distributions of both local CSFEs and local APBEs.

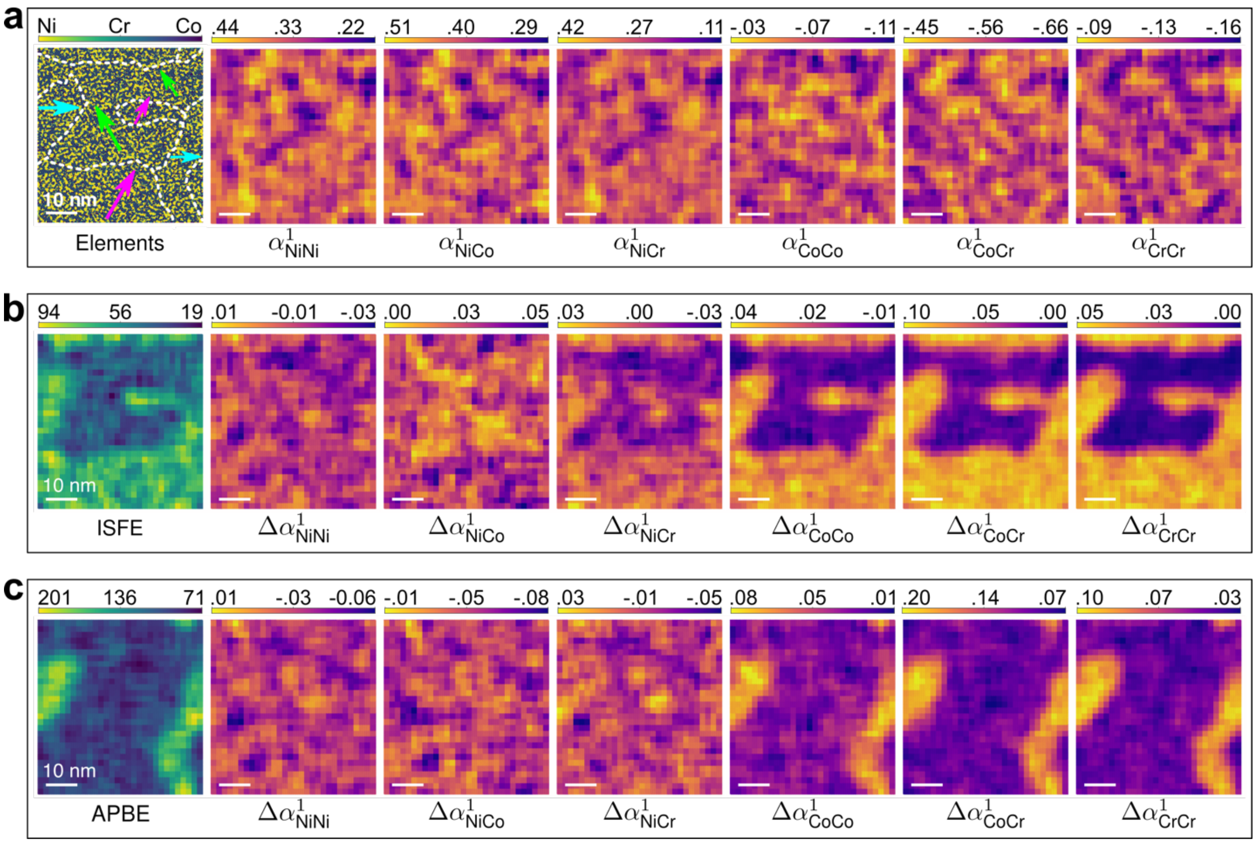

Supplementary Figure 12 | Nano-scale heterogeneities and their correlations. The LCO in this sample was developed at T_a = 650 K. **a**, Element distribution and the spatially varying local chemical order parameter. Arrows with different colors in the leftmost figure denote the orientations of the Co-Cr domains. Dashed lines are domain boundaries. **b**, Spatial distribution of local complex stacking fault energies and the corresponding changes of local chemical orders ($\Delta\alpha_{ij}^{1}$) due to the gliding of a leading partial dislocation. **c**, Spatial distribution of local anti-phase boundary energies and the corresponding changes of local chemical orders ($\Delta\alpha_{ij}^{1}$) caused by the gliding of a full dislocation. $\Delta\alpha^{1} = \Delta\alpha^{1,\text{ISF}} - \Delta\alpha^{1,\text{perfect}}$ for (**b**) and $\Delta\alpha^{1} = \Delta\alpha^{1,\text{APB}} - \Delta\alpha^{1,\text{perfect}}$ for (**c**). The value of each local area is calculated based on a fault area of 3.2 nm^2 and averaged over its 1st and 2nd nearest neighbor areas (9 areas in total including the central area). The scale bar is 10 nm.

Specifically, regions with smaller $\Delta\alpha_{\text{CoCo}}^{1}$, $\Delta\alpha_{\text{CoCr}}^{1}$ and $\Delta\alpha_{\text{CrCr}}^{1}$ show lower CSFEs and APBEs, and vice versa (Supplementary Figure 12b-c). Furthermore, the local CSFE (Supplementary Figure 12b) and local APBE (Supplementary Figure 12c) distributions coincide with certain Co-Cr domains shown in Supplementary Figure 12a. For example, the relatively dark area in local CSFE mapping (Supplementary Figure 12b) corresponds to the green-arrow domain in Supplementary Figure 12a, while the darker region in local APBE mapping (Supplementary Figure 12c) matches the area occupied by the magenta and green-arrow domains in Supplementary Figure

12a. This suggests that the local fault energies are highly sensitive to the orientations of Co-Cr domains, in addition to the degree of Co-Cr LCO. For samples with much higher T_a, the orientation sensitivity would decrease as the Co-Cr clusters are too randomly oriented to form well-defined domains; the fault energy is more closely related to the degree of Co-Cr chemical order. The local fault energies only show weak correlations to Ni-related chemical order changes (Supplementary Figure 12b-c). This is because FCC Ni precipitates are isotropic with respect to the <112> shear. Thus each of them may experience similar chemical order changes after shearing, and the whole region looks more uniform than non-Ni chemical order changes (i.e., $\Delta\alpha^1_{\mathrm{CoCo}}$, $\Delta\alpha^1_{\mathrm{CoCr}}$ and $\Delta\alpha^1_{\mathrm{CrCr}}$).

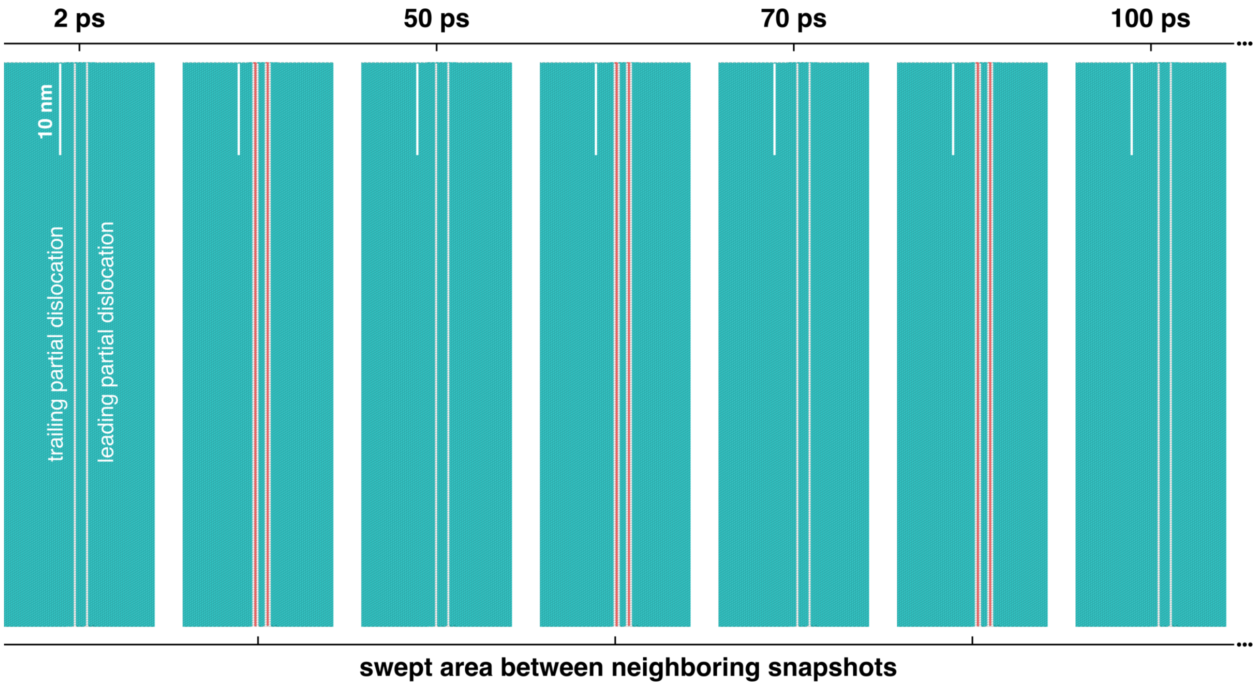

Supplementary Figure 13 | Uniform and smooth glide of extended screw dislocation in Cu. The initial dislocation configuration is shown by the snapshot at 2 ps. This extended screw dislocation is then subjected to a constant shear stress of ~ 10 MPa at 300 K. The configurations at 50 ps, 70 ps and 100 ps demonstrate a forward glide process, with the uniform swept areas highlighted in red. All configurations are obtained by quenching the MD configurations to zero K and zero stress and energy minimization.

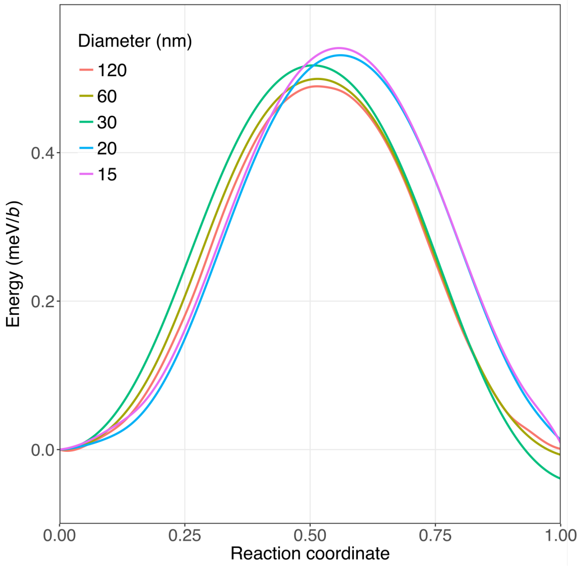

Supplementary Figure 14 | Sample size (cylinder diameter) effect on the Peierls barrier. The calculations are based on cylinder configurations, using an EAM potential for Al[15]. All cylinder configurations have an axial length of 4b, where b is the magnitude of the full Burgers vector. A screw dislocation (with **b** along the axial direction) was introduced into the cylinder configuration following the anisotropic elasticity theory. Then atoms belonging to the outer layer with a thickness greater than two times the potential cutoff are fixed during the subsequent energy minimization. The free-end string method[16] was used for the minimum energy path calculations. As seen, with smaller diameters, the Peierls barrier only slightly increases, showing a limited size effect on the barriers. Likewise, since we used the same size for all the samples in our main text, the sample size should have a negligible effect on the relative magnitude of the Peierls barriers evaluated for samples with different T_a.

Supplementary Note 5

We carried out the minimum energy path (MEP) calculations in samples under different processing conditions including RSS, T_a = 1350 K, T_a = 950 K and T_a = 650 K. For each processing condition, we used 30 different samples under different stress levels to collect sufficient data points for statistical comparison. Supplementary Figure 15 shows the effective activation barriers for all types of samples. From the plot, several important observations can be made. First, at a specific local stress level, the activation barrier is not a single value; instead the magnitude of the activation barrier spans a wide range. Such a spread of activation barriers results from the nanoscale heterogeneities as shown in Fig. 3 in the main text. Second, for a given stress level, increasing LCO (RSS → T_a = 1350 K → T_a = 950 K → T_a = 650 K) leads to an increasingly wider range of barrier heights, suggesting additionally higher barriers in the more ordered samples. Third, for samples processed at each T_a, the barrier range gradually narrows with increasing local shear stress,

approaching a vanishing barrier. Such barrier-range expansion and athermal stress limit increase with increasing LCOs suggest remarkable LCO-induced strengthening as discussed in the main text.

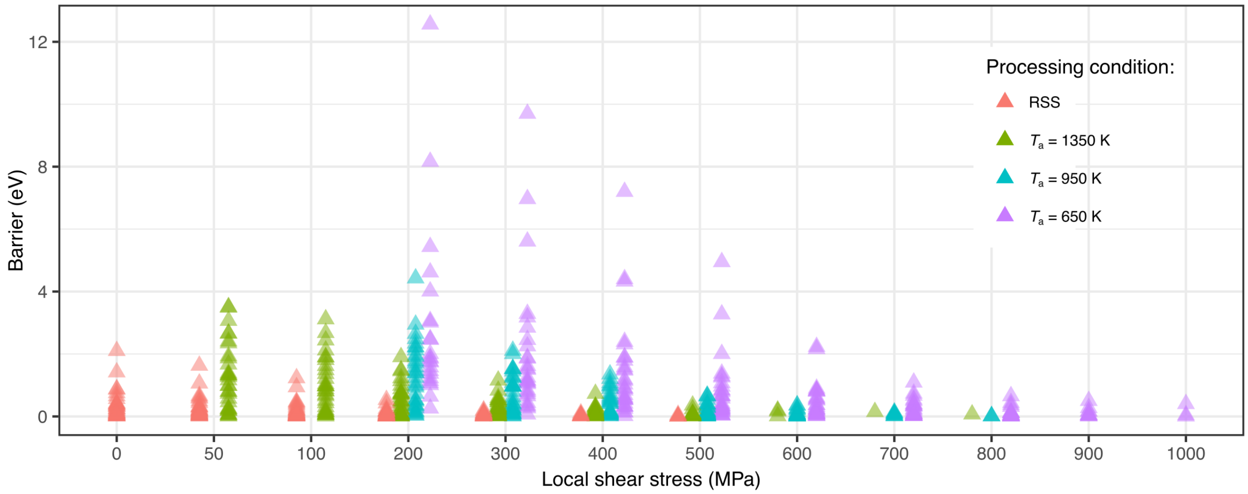

Supplementary Figure 15 | Effective barriers calculated for nanoscale depinning processes in different samples.

Supplementary Note 6

The activation volumes at finite temperatures can be obtained from the stress dependence of the activation free energies, $\Omega(\tau, T) = -\partial Q(\tau, T)/\partial\tau$. Following previous work[3,17,18], the activation free energy is approximated as $Q(\tau, T) = Q_0\left(1 - \frac{T}{T^*}\right)\left(1 - \frac{\tau}{\tau_{\mathrm{ath}}}\right)^{\alpha}$, where Q_0 is the activation energy at zero-temperature and zero-stress, τ_{ath} is the athermal strength, T^* is a characteristic temperature for estimating the activation entropy and α is a constant controlling the stress dependence of Q. The characteristic temperature T^* is evaluated as $\frac{1}{T^*} = \frac{1}{\mu_0}\frac{\mathrm{d}\mu}{\mathrm{d}T}$, where μ_0 is the zero-temperature shear modulus and $\frac{\mathrm{d}\mu}{\mathrm{d}T}$ is the changing rate of shear modulus with respect to temperature. For each type of sample, we calculated the shear modulus at 0 K, 50 K, 100 K, 150 K, 200 K, 250 K and 300K, respectively. At each temperature, we carried out ten calculations to obtain the statistical mean shear modulus. Then $\frac{\mathrm{d}\mu}{\mathrm{d}T}$ can be obtained by considering all the temperature-dependent shear modulus. Finally, the T^* for different samples were obtained as follows: 6909 K, 7653 K, 8973 K and 8446 K, for samples of RSS, T_a = 1350 K, T_a = 950 K, T_a = 650 K, respectively. Note that T^* for samples with T_a = 650 K is slightly lower than that for samples

with T_a = 950 K. This is consistent with the results in Supplementary Figure 11, where a temporary shear modulus softening shows up during the significant local chemical ordering process around T_a = 850 K. The reason for this shear modulus softening was explained in Supplementary Note 3.

The intrinsic yielding strength can then be evaluated by solving Orowan's equation

$$\dot{\gamma} = \rho_{\mathrm{m}} b \bar{v} = \rho_{\mathrm{m}} b v_0 \bar{d} \exp\left(-\frac{Q(\tau,T)}{k_{\mathrm{B}}T}\right) \quad (2)$$

where ρ_{m} is the mobile dislocation density (~10^8 m^{-2}, assuming one dislocation in a grain with typical grain size of 100 μm), $\bar{v}$ is the average dislocation velocity which can be further expressed in the form of $\bar{v} = v_0 \bar{d} \exp\left(-\frac{Q(\tau,T)}{k_{\mathrm{B}}T}\right)$, with v_0 the attempt frequency (~10^{12} s^{-1}, see Supplementary Note 8), $\bar{d}$ the average distance a dislocation segment moves through an activation event (~1.1 nm, an average value over 50 events in different samples under different stresses), and k_{B} the Boltzmann's constant.

Supplementary Note 7

Edge dislocations were simulated using the equilibrium configurations from the hybrid MD and MC simulations. The as-prepared sample was first replicated along $y[111]$ direction by 2 times and then replicated along $z[1\bar{1}0]$ direction by 2 times. As schematically illustrated in Supplementary Figure 16, the simulation box has a geometry of $X[11\bar{2}]$ 52 nm × $Y[111]$12.5 nm × $Z[1\bar{1}0]$ 111 nm. An edge dislocation was introduced according to the following procedure. First, we centered the desired slip plane to the center of the simulation box and turned off the periodic boundary conditions along y direction. Then we divided the simulation box into upper and lower slabs. For the lower slab, an atomic plane perpendicular to the $z[1\bar{1}0]$ direction was deleted. In this way, an extra plane was introduced in the upper slab. Atoms beside the deleted plane were rescaled to fill up the extra space. Then the simulation box was uniformly scaled such that the z direction length was reduced by half of the Burgers vector. Energy minimization was further performed to relax the defect configuration, after which the as-introduced dislocation configuration was relaxed at 300 K for 100 ps and then the temperature was increased to 1000 K to relax for an extra100 ps. The high-temperature relaxed configuration was then cooled down to 300 K and the boundary condition along the dislocation motion direction was also changed to free surface. Finally, after a short period of relaxation at 300 K, a constant shear strain rate of 1×10^7 s^{-1} was applied by assigning a constant velocity in z direction to the top surface atoms (in y direction) while the bottom surface atoms were fixed. The introduced dislocation is ~52 nm long to allow sufficient variations of core configurations along the line sense direction. The relaxed simulation configuration is schematically illustrated in Supplementary Figure 16.

As shown in Supplementary Figure 17, the full dislocation generally dissociates into partial dislocations with variable curvatures along dislocation line sense direction. The dissociation widths in samples of higher T_as are generally larger than those of relatively lower T_as. For random solid solutions, the separation between partial dislocations becomes significantly large after relaxation at 1000 K, which is no longer appropriate for the subsequent shear deformation. Instead, the configuration shown in the left panel of Supplementary Figure 17a was used to perform the shear deformation. The dissociation width in samples of T_a = 1650 K remains largely constant before and after relaxation at 1000 K. The dissociation width in samples of both T_a = 1350 K and T_a = 950 K becomes smaller after relaxation at 1000 K. These results demonstrate that both the lattice resistance and the CSFE affect the dislocation dissociation width. For samples of T_a = 650 K and T_a = 350 K, due to the large APBE and CSFE, the as-introduced dislocation is already able to overcome the lattice resistance to form relatively narrow dislocation cores which largely remain constant after relaxation at 1000 K. Overall, the dislocation core configurations, in terms of the variation along the dislocation line and dissociation behavior, are strongly dependent on T_a and thus local chemical ordering.

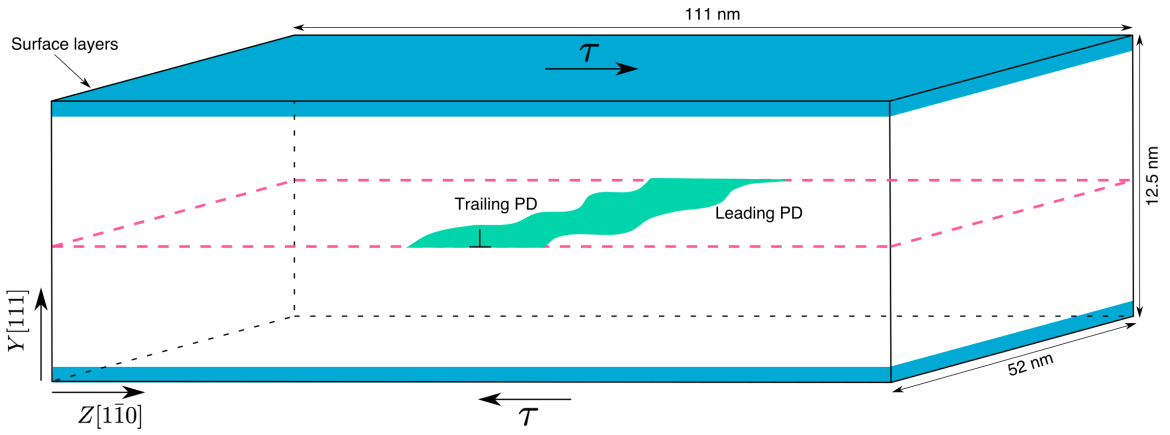

Supplementary Figure 16 | Schematic illustration of the simulation setup for edge dislocations. Periodic boundary conditions are applied in dislocation line direction (i.e., X direction), while free surfaces are used in both Y and Z directions. The introduced edge dislocation generally dissociates into two partial dislocations (PD) with wavy dislocation lines. To apply a constant strain rate, a constant velocity in the Z direction was assigned to the top surface atoms (region colored in blue) while the bottom surface atoms were fixed.

Supplementary Figure 18 shows the shear stress vs. time curve (middle panel) and the corresponding snapshots of dislocations in samples with T_a = 650 K (upper panel) and T_a = 1350 K (lower panel), respectively. A significant stress drop on the curve corresponds to the movement of a partial dislocation. Several interesting phenomena arise from the shear responses of these dislocations.

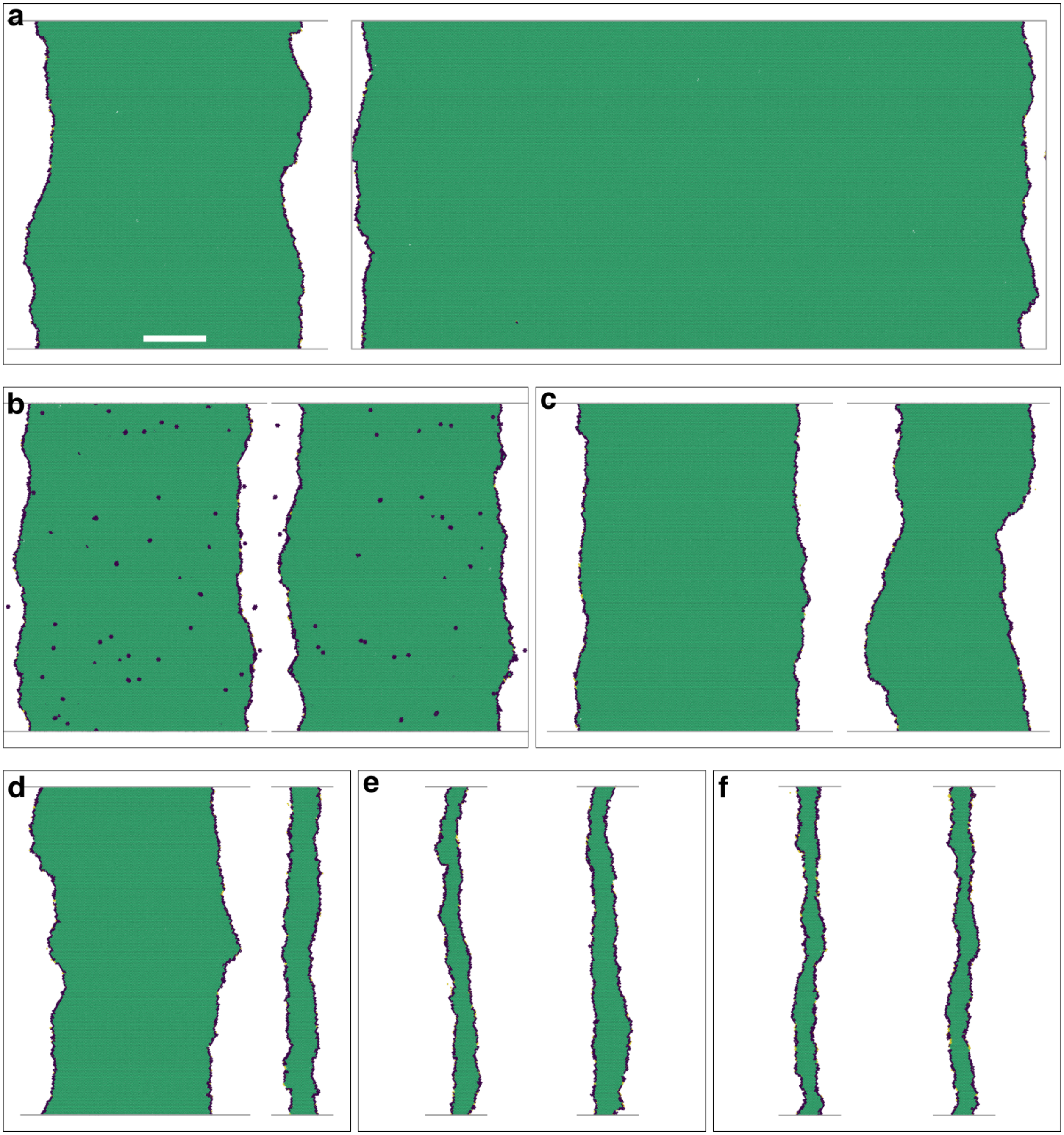

Supplementary Figure 17 | Dislocation core configurations for random sample (**a**), sample annealed at 1650 K (**b**), sample annealed at 1350 K (**c**), sample annealed at 950 K (**d**), sample annealed at 650 K (**e**) and sample annealed at 350 K (**f**). In each figure, the left configuration is the as-introduced dislocation core after relaxation at 300 K for 100 ps and the right configuration is after relaxation at 1000 K for 100 ps and then cooled down to 300 K. The scale bar is 10 nm. Atoms in stacking fault are rendered in green while atoms in dislocation core or point defects are in purple. The small atom clusters in (**b**) are formed by thermally induced point defects due to annealing at temperatures not far from the melting point.

First, as T_a decreases, a significant increase in the resistance to both the leading partial dislocation and the trailing partial dislocation is observed. Specifically, for samples of random solid solution, T_a = 1350 K, 950 K and 650 K, the critical shear stresses for the leading partial dislocation are 0.35 GPa, 0.52 GPa, 0.64 GPa and 1.10 GPa, respectively. The strengthening is also seen in the increasing critical shear stress to drive the trailing partial dislocation, 0.60 GPa, 0.80 GPa, 1.00 GPa and 0.92 GPa, respectively. Such pronounced strengthening effects can be attributed to the increasing LCOs with decreasing T_a: it costs more energy for a partial dislocation to break loose from the increasingly stronger LCOs as T_a decreases, consistent with the results on CSFE and APBE in the main text.

Second, the atomic scale LCO and the resultant nanoscale segregations/clusters act as strengthening obstacles to dislocation motion. Indeed, dislocation lines frequently show remarkable local curvatures during motion (see Supplementary Movie 1-2 for dynamic processes, for T_a= 1350 K and T_a = 650 K), which indicates strong impedance of local dislocation segments that would otherwise move rather smoothly. Such a rugged advancing dislocation line is consistent with the abundant nanoscale heterogeneities shown in both Fig. 1 and Fig. 3 in the main text, much like sailing in the choppy "sea" of LCO. The extra resistance leads to an elevated Peierls stress. The heterogeneous dislocation motion may create significant line tension modifying the magnitude of the local stress. In this case, it is not sufficient to use the externally applied stress to specify the stress-sensitive local activation barriers; the local stress dependences of the activation barriers shown in Fig. 5a are thus more intrinsic.

Third, the trailing partial dislocation is much harder to move than the leading partial dislocation. For example, for the random solution and the $T_a \geq 950$ K samples, the critical shear stress to drive a trailing partial dislocation is ~60% higher than that to drive a leading partial dislocation. Such higher stresses to drive trailing partial dislocation is not due to our simulation setup. Specifically, the current simulation box has a large in-plane size (Supplementary Figure 16) of 52 nm (dislocation line direction) × 111 nm (dislocation motion direction), so the box size effects should have been minimal. Instead, the higher stresses needed to drive the trailing partial dislocations are caused by the relatively larger energy cost to eliminate the complex stacking fault (CSF) and create local antiphase boundaries (APB). This can be seen from differences between APBE and CSFE, i.e., APBE – CSFE is a larger value when compared to CSFE. For example, for T_a = 1350 K and T_a = 950 K, the differences between APBE and CSFE are 52.24 mJ/m^2 and 58.37 mJ/m^2, respectively, while their CSFEs are only 9.11 mJ/m^2 and 22.58 mJ/m^2, respectively. Thus, the average energy penalty to eliminate CSF is considerably higher than that to create CSF, resulting in higher stresses to drive trailing partial dislocations. In contrast, for samples with T_a = 650 K, APBE – CSFE is 60.60 mJ/m^2 while the CSFE is 60.69 mJ/m^2, thus the stresses needed to drive leading partial dislocation and trailing partial dislocation should be comparable to each other. However, for samples with such lower T_a, the stresses to drive leading and trailing partial

dislocation would also depend on the local Co-Cr domain orientations (see Supplementary Figure 12).

The more energetically favorable motion of the leading partial dislocation should significantly enhance the formation of uniformly distributed SFs and a high population of very thin (nano-)twins. Indeed, profuse SFs and nanoscale twins were reported to be responsible for the strong work hardening and good ductility of the HEAs/MEAs[19–21,2,22]. Experiments also reported the preference for planar slip in HEAs[19,20,23–29], which would be expected from repeated operation of dislocation that eliminates the LCO on some specific planes (see Fig. 2 in main text). In other words, the plethora of experimental observations in HEAs/MEAs, including the local SFE, faults and nanotwins versus extended dislocations, planar slip, and the very different strength and hardening behavior for HEA/MEA samples processed at different annealing or homogenization temperatures, can now all be explained under the same umbrella of the variable LCO.

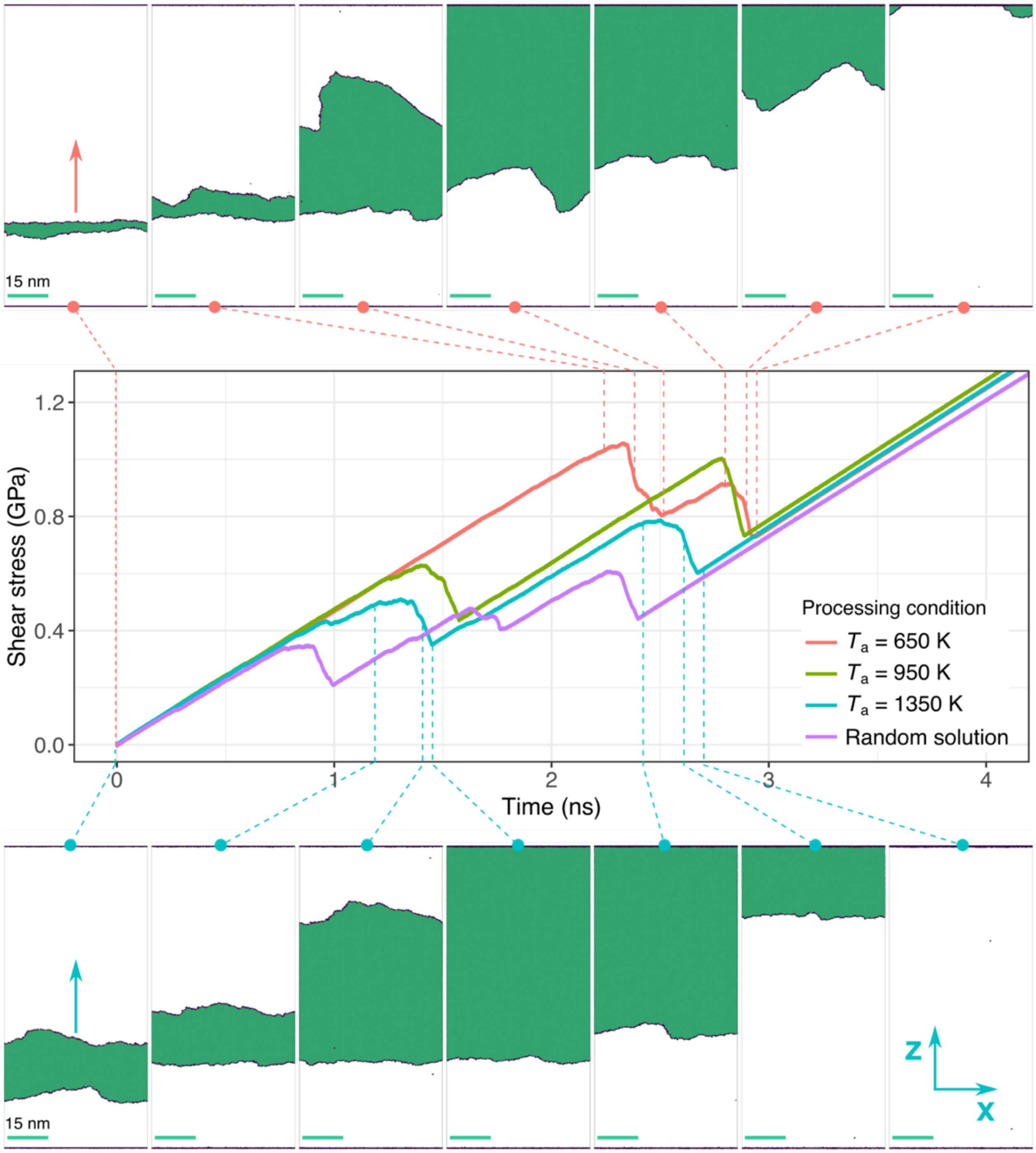

Supplementary Figure 18 | Local chemical order induces strengthening. Dislocations in the random solution sample and samples annealed at 1350 K, 950 K and 650 K are subjected to simple shear at 300 K with a constant shear strain rate of 1×10^7 s^{-1}. The middle panel shows the shear stress vs. time curve, showing the strengthening due to increasing LCO that triples the stress needed to move the dislocation. The upper and lower panels show snapshots of dislocation configurations in samples annealed at 650 K and 1350 K, respectively. The arrows indicate the motion direction of the dislocation. X is along the $[11\bar{2}]$ direction and Z is along the $[1\bar{1}0]$ direction. The scale bar in each snapshot is 15 nm.

Supplementary Note 8

For minimum energy path calculations, we followed the suggestions made by Nöhring and Curtin[30] that the string method could be more robust for complex concentrated alloys. However, we did compare the string method results with NEB calculations. Supplementary Figure 19 shows an example of the MEPs calculated by string method and NEB method, respectively. As can be seen, the overall profiles of both MEPs are very similar to each other; those small differences should be due to the specific constraints used in updating intermediate replicas (e.g., numerical reparameterization for string method vs. nudged potential forces for NEB method). Despite these slight differences, the major saddle points from both methods coincide with each other very well, suggesting very good consistency. We also extensively tested the convergence criteria used in the string method. Overall, results obtained using the current criteria are comparable to well converged NEB calculations. For example, in Supplementary Figure 19, the NEB calculation was considered as converged only when the forces on each replica are less than 0.001 eV/Å.

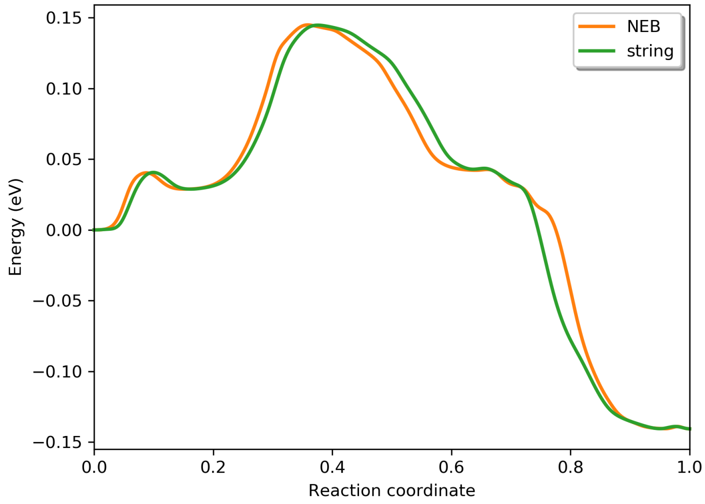

Supplementary Figure 19 | Comparison between the minimum energy paths calculated using the string method and the NEB method. This example corresponds to Fig. 4b in the main text.

It should be noted that for complex rugged energy landscape, there may be multiple transition pathways between the same set of initial/final states. The calculated MEP from either string method or NEB method is only one possible transition path; more advanced methods (e.g., finite-temperature string method and forward flux sampling etc.) are required to obtain the accurate transition rate of a specific event. However, in this work, we are not focusing on a single event but rather on sampling possible nanoscale segment detrapping events in the entire sample. For this purpose of the current work, string method calculations/NEB calculations over many randomly chosen events would draw a sample set that reflects the characteristics of the entire distribution. Such calculated barriers can be further verified using Arrhenius plot. For example, Supplementary

Figure 20 plots the natural logarithm of partial dislocation velocities (ln (v)) against $1/(k_B T)$ in RSS samples subjected to a shear stress of 200 MPa. As can be seen, the fitted average activation energy in this case is 0.052 eV which is close to the string-method calculated activation energy 0.085 eV (with a standard deviation of 0.0625 eV). On the other hand, the fitted intercept ln (A), where $A = v_0 \bar{d} \exp(\Delta S)$, is 7.8. As we already know $\bar{d}$ and exp (ΔS) (from the thermodynamic compensation rule in Supplementary Note 6), the attempt frequency is estimated to be $2.1 \times 10^{12} \mathrm{s}^{-1}$, which is in the expected range from $10^{11} \mathrm{s}^{-1}$ to $10^{13} \mathrm{s}^{-1}$.

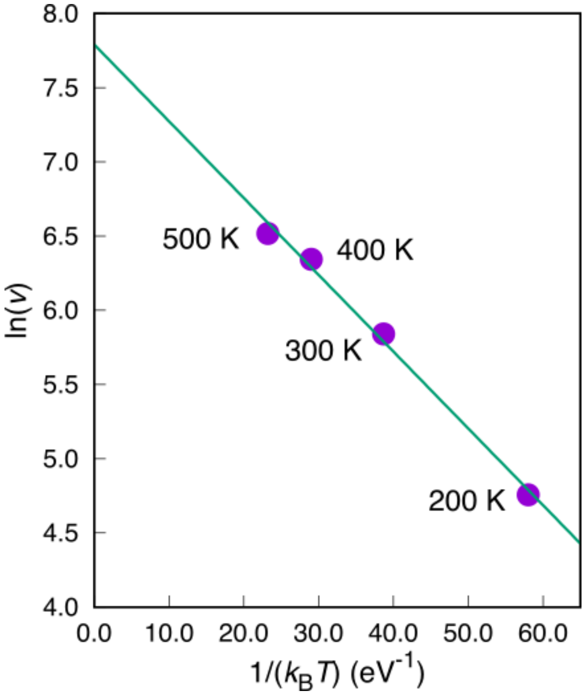

Supplementary Figure 20 | Arrhenius plot of partial dislocation velocities (ln (v)) vs. $1/(k_B T)$ in RSS. The applied shear stress is 200 MPa. The dislocation line length is ~30 nm and the glide distance is ~64 nm. Each data point was averaged over 5 different RSS samples. The Arrhenius-plot-informed average activation energy is 0.052 eV (the negative slope) which is close to the string-method calculated activation energy 0.085 eV with a standard deviation of 0.0625 eV.

Supplementary Note 9

There have been no well-accepted potentials so far for multi-component HEAs (the published MD simulation papers had to use some average atom potentials), seriously hampering atomistic modeling of these new concentrated alloys. As detailed in Supplementary Note 1, our potential was developed based on non-magnetic DFT calculations and experimental inputs. Spin polarization was not explicitly considered. Thus, our potential is an empirical potential to model the non-magnetic Ni-Co-Cr system and it is not intended for situations with significant magnetic effects (although some magnetic effects are implicitly incorporated from input properties of pure elements). In other words, the as-developed EAM potential is a highly optimized atomistic model

for equi-atomic multicomponent system, the best there is for HEAs; however, it is not "realistic" to the point that it can describe magnetic transition and spin polarization effects in detail with accuracy.

In fact, atomistic models employing classic potential formalisms (e.g., the embedded-atom-method adopted here) never fully account for the electron spins. This is because of i) the limitation of the potential formalism: the goal is the construction of a potential that allows handling of large-scale simulations; but this is achieved at the expense of the accuracy of first-principles calculations. ii) The level of difficulty in accurately describing the magnetic states of the system. In practice, the magnetic effect is usually not explicit in such classic MD potentials and simulations. Even for pure Ni, with a Curie temperature of ~627K, MD simulations do not have the predictive power for its magnetic transition.

In general, atomistic models developed using the force-matching method will not be as accurate as DFT calculations. In our development of the Ni-Co-Cr potential, despite of our best effort, the average energy difference between the DFT data and the EAM potential is ~20 meV/atom (see Supplementary Note 1). In the field of potential development, this margin of deviation is normally already considered a highly optimized interatomic potential for atomistic MD modeling. But the empirical potential could fail to capture the magnetic effects, if the resultant energy difference is only several meV/atom. This is essentially inherent to the empirical potential development, rather than our potential fitting procedure itself.

Then the next question is how large are the magnetic effects in NiCoCr. We start our discussion from the random solution at this composition. Our spin-polarized DFT calculation shows a very small energy difference between the magnetic phase and the non-magnetic fcc phase, 2 meV/atom, and that between magnetic and non-magnetic hcp states is around 4 meV/atom (see Supplementary Figure 21 and its caption for methods). The SFE of the magnetic state is slightly more negative than the non-magnetic state by a difference of ~ 5-10 mJ/m^2. Our present model can capture the energy difference between HCP and FCC NiCoCr random solid solutions. This is discussed in the context of intrinsic stacking fault energy in the main text. Our potential was optimized to reproduce the negative stacking fault energy of NiCoCr even without explicitly considering the magnetic effect. Both our calculations and Niu's work[4] clearly indicate that the HCP phase is favored over the fcc phase with or without magnetic contributions (see Supplementary Figure 21) in the equiatomic NiCoCr alloy. In the CrMnFeCoNi alloy, the magnetic effect is complicated by the presence of Mn atoms (due to strong magnetic frustrations of Mn). In other words, the CrMnFeCoNi alloy has a much stronger magnetism effect, as opposed to the NiCoCr alloy. Our atomistic model for NiCoCr yields similar behavior as the DFT calculations.

Experimentally, according to the report from an Oak Ridge group in Scientific Reports[31] in 2016, the antiferromagnetism of Cr frustrates the ferromagnetism of NiCo. The magnetic ordering does not show up at this composition all the way down to 2 K. The system behaves like a paramagnetic material with a susceptibility like Pd metal.

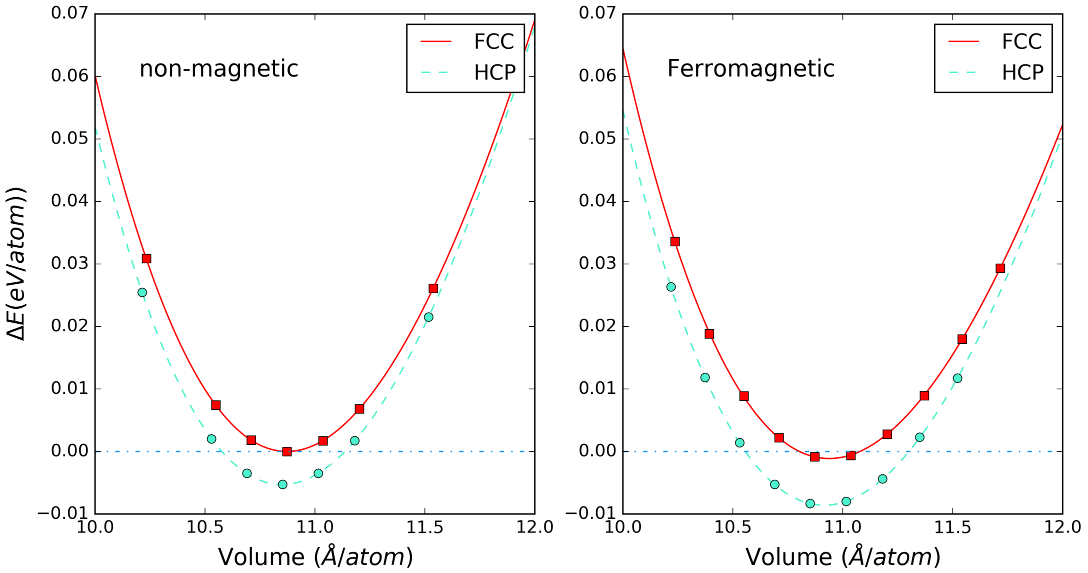

Supplementary Figure 21 | Results of ab initio calculations of the energy differences between fcc and hcp NiCoCr at 0K. Equation of states of fcc and hcp CrCoNi phases in the non-magnetic (left panel) and the ferromagnetic (right panel) states. The energies are plotted with reference to the non-magnetic fcc NiCoCr structure at the ground state. In both cases, the hcp structure is found to have a smaller formation energy. The magnetic contribution is found to be small in the NiCoCr alloy. The randomly populated super-cell approach was used to obtain the formation energies of fcc and hcp NiCoCr alloys, both in the magnetic state and in the non-magnetic state. The formation energies were averaged over 20 random configurations. For each calculation, the configuration (360 atoms) was geometrically optimized to reach the ground state at 0K.

But this 2016 experimental measurement also indicates that when the Cr content is lowered, magnetic ordering does become more obvious. When the alloy composition shifts to NiCoCr0.5 (Curie temperature rises to 250 K), our spin-polarized DFT calculations show that the energy difference between the magnetic and non-magnetic states becomes 15 meV/atom (and the volume difference is less than 2%). Therefore, even in this “obviously magnetic” case, the energy difference between the spin-polarized and non-polarized states is still relatively low, not up to the level that an EAM potential can resolve (~20 meV/atom, see Supplementary Note 1). As such, the magnetic effects may increase in our modeling when our solution alloy develops increasing local (partial) chemical order at low ageing temperatures. For example, we performed spin-polarized DFT calculations on the configurations (each 360 atoms) with various LCOs (Supplementary Figure 7), and found that the configuration with the largest compositional variations shows an energy difference that is similar to that of the NiCoCr0.5 case above. Magnetic effects with such

magnitude may bring some impact on the LCO and the magnitude of the order parameters, especially if the magnetic effects induced energy difference is comparable or larger than that due to chemical ordering.

We mention here that the mechanical behavior of interest is mostly in the temperature range from room-temperature down to liquid nitrogen temperature. The zero-K potential energy landscape we describe is actually the finite-temperature free energy landscape with the thermal contribution subtracted from it. This energy landscape is not really the one at 0 K when some ferromagnetic state prevails, as finite temperature would randomize most of the spins.

All these said, we still have to admit that fundamentally, the EAM formalism is incapable of capturing complex magnetic effects at finite temperatures in a self-consistent manner. Some type of spin-dependent potential needs to be developed, but is beyond reach at present. As mentioned earlier, thus far EAM potentials have never been meant to monitor various degrees of magnetic ordering.

Therefore, the EAM potential as developed is meant to be an empirical NiCoCr atomistic model that enables a parametric study of the trend of dislocation behavior. This atomistic model is designed to capture the typical features of HEAs and MEAs: multi-principal (equiatomic) constituents, moderate chemical interactions, similar atomic sizes, single phase but with variable local chemical order, etc. The model is meant to analyze the trend due to LCO and consequences on dislocation responses, rather than pinning down energy numbers or nailing down the various contributions from chemical, elastic or magnetic energy terms. The model still overcomes a major hurdle in atomistic studies of high-entropy alloys: it allows large-scale (e.g., multi-million atoms simulations) MD modeling of dislocation activities, outside the realm of the first-principles DFT calculations.

Supplementary References

1. Kittel, C. *Introduction to Solid State Physics*. (Wiley, 2004).

2. Zhang, Z. *et al.* Dislocation mechanisms and 3D twin architectures generate exceptional strength-ductility-toughness combination in CrCoNi medium-entropy alloy. *Nat. Commun.* **8**, 14390 (2017).

3. Ryu, S., Kang, K. & Cai, W. Predicting the dislocation nucleation rate as a function of temperature and stress. *J. Mater. Res.* **26**, 2335–2354 (2011).

4. Niu, C., LaRosa, C. R., Miao, J., Mills, M. J. & Ghazisaeidi, M. Magnetically-driven phase transformation strengthening in high entropy alloys. *Nat. Commun.* **9**, 1363 (2018).
5. Alam, A., Chouhan, R. K. & Mookerjee, A. Phonon modes and vibrational entropy of disordered alloys with short-range order: A first-principles calculation. *Phys. Rev. B* **83**, 054201 (2011).
6. Körmann, F., Ikeda, Y., Grabowski, B. & Sluiter, M. H. F. Phonon broadening in high entropy alloys. *Npj Comput. Mater.* **3**, 36 (2017).
7. Erhart, P., Caro, A., Serrano de Caro, M. & Sadigh, B. Short-range order and precipitation in Fe-rich Fe-Cr alloys: Atomistic off-lattice Monte Carlo simulations. *Phys. Rev. B* **77**, 134206 (2008).
8. Wang, Y., Zacherl, C. L., Shang, S., Chen, L.-Q. & Liu, Z.-K. Phonon dispersions in random alloys: a method based on special quasi-random structure force constants. *J. Phys. Condens. Matter* **23**, 485403 (2011).
9. Dove, M. T. *Introduction to lattice dynamics*. (Cambridge University Press, 2007).
10. Hellman, O., Steneteg, P., Abrikosov, I. A. & Simak, S. I. Temperature dependent effective potential method for accurate free energy calculations of solids. *Phys. Rev. B* **87**, 104111 (2013).
11. Tadano, T., Gohda, Y. & Tsuneyuki, S. Anharmonic force constants extracted from first-principles molecular dynamics: applications to heat transfer simulations. *J. Phys. Condens. Matter* **26**, 225402 (2014).
12. Khachaturyan, A. G. Ordering in substitutional and interstitial solid solutions. *Prog. Mater. Sci.* **22**, 1–150 (1978).

13. Bonny, G. *et al.* The influence of short range order on the thermodynamics of Fe–Cr alloys. *Model. Simul. Mater. Sci. Eng.* **17**, 025006 (2009).

14. Ustinovshikov, Y. Phase transformations in alloys of the Ni–Cr system. *J. Alloys Compd.* **543**, 227–232 (2012).

15. Zope, R. R. & Mishin, Y. Interatomic potentials for atomistic simulations of the Ti-Al system. *Phys. Rev. B* **68**, 024102 (2003).

16. Kang, K. Atomistic Modeling of Fracture Mechanisms in Semiconductor Nanowires under Tension. (Stanford University, 2010).

17. Li, Q.-J., Xu, B., Hara, S., Li, J. & Ma, E. Sample-size-dependent surface dislocation nucleation in nanoscale crystals. *Acta Mater.* **145**, 19–29 (2018).

18. Zhu, T., Li, J., Samanta, A., Leach, A. & Gall, K. Temperature and Strain-Rate Dependence of Surface Dislocation Nucleation. *Phys. Rev. Lett.* **100**, 25502 (2008).

19. Zhang, Z. *et al.* Nanoscale origins of the damage tolerance of the high-entropy alloy CrMnFeCoNi. *Nat. Commun.* **6**, 10143 (2015).

20. Gludovatz, B. *et al.* A fracture-resistant high-entropy alloy for cryogenic applications. *Science* **345**, 1153–1158 (2014).

21. Gludovatz, B. *et al.* Exceptional damage-tolerance of a medium-entropy alloy CrCoNi at cryogenic temperatures. *Nat. Commun.* **7**, 10602 (2016).

22. Xu, X. D. *et al.* Transmission electron microscopy characterization of dislocation structure in a face-centered cubic high-entropy alloy Al0.1CoCrFeNi. *Acta Mater.* **144**, 107–115 (2018).

23. Otto, F. *et al.* The influences of temperature and microstructure on the tensile properties of a CoCrFeMnNi high-entropy alloy. *Acta Mater.* **61**, 5743–5755 (2013).

24. Laplanche, G., Kostka, A., Horst, O. M., Eggeler, G. & George, E. P. Microstructure evolution and critical stress for twinning in the CrMnFeCoNi high-entropy alloy. *Acta Mater.* **118**, 152–163 (2016).

25. Zhao, Y. L. *et al.* Heterogeneous precipitation behavior and stacking-fault-mediated deformation in a CoCrNi-based medium-entropy alloy. *Acta Mater.* **138**, 72–82 (2017).

26. Miao, J. *et al.* The evolution of the deformation substructure in a Ni-Co-Cr equiatomic solid solution alloy. *Acta Mater.* **132**, 35–48 (2017).

27. Deng, Y. *et al.* Design of a twinning-induced plasticity high entropy alloy. *Acta Mater.* **94**, 124–133 (2015).

28. Smith, T. M. *et al.* Atomic-scale characterization and modeling of 60° dislocations in a high-entropy alloy. *Acta Mater.* **110**, 352–363 (2016).

29. Yasuda, H. Y., Shigeno, K. & Nagase, T. Dynamic strain aging of Al0.3CoCrFeNi high entropy alloy single crystals. *Scr. Mater.* **108**, 80–83 (2015).

30. Nöhring, W. G. & Curtin, W. A. Dislocation cross-slip in fcc solid solution alloys. *Acta Mater.* **128**, 135–148 (2017).

31. Sales, B. C. *et al.* Quantum Critical Behavior in a Concentrated Ternary Solid Solution. *Sci. Rep.* **6**, 26179 (2016).
