# Supplementary material for: Strengthening in multi-principal element alloys with local-chemical-order roughened dislocation pathways
Source: Nat Commun. 2019 Aug 8;10:3563. doi: 10.1038/s41467-019-11464-7 (PMC6687833; doi:10.1038/s41467-019-11464-7)
Supplement: Supplementary file 3 — Description of Additional Supplementary Files [file 41467_2019_11464_MOESM3_ESM.docx]

**Description of Supplementary Files**

**File Name:** **Supplementary Movie 1**

**Description:** Dynamic motion of an extended edge dislocation in a sample processed at Ta = 1350 K.

**File Name:** **Supplementary Movie 2**

**Description:** Dynamic motion of an extended edge dislocation in a sample prepared at Ta = 650 K.
